# Antioxidant and Antimycotic Activities of Two Native *Lavandula* Species from Portugal

**DOI:** 10.1155/2015/570521

**Published:** 2015-04-01

**Authors:** Rafael Baptista, Ana Margarida Madureira, Rita Jorge, Rita Adão, Aida Duarte, Noélia Duarte, Maria Manuel Lopes, Generosa Teixeira

**Affiliations:** ^1^Research Institute for Medicines (iMed.ULisboa), Faculdade de Farmácia, Universidade de Lisboa, Avenida Professor Gama Pinto, 1649-003 Lisboa, Portugal; ^2^Universidade de Lisboa, Instituto Superior de Agronomia, Tapada da Ajuda, 1349-017 Lisboa, Portugal; ^3^Universidade de Lisboa, Faculdade de Farmácia, Centre for Ecology, Evolution and Environmental Changes, (Ce3C), Avenida Professor Gama Pinto, 1649-003 Lisboa, Portugal

## Abstract

The antioxidant and antimycotic activities of the essential oils and extracts of two native Portuguese *Lavandula* species, *L. stoechas* subsp. *luisieri* and *L. pedunculata*, were evaluated by *in vitro* assays. The total phenolics and flavonoids content were also determined. The antioxidant potential was assessed through DPPH radical scavenging, inhibition of lipid peroxidation (ILP), and DNA protection assays. All samples displayed a high DPPH scavenging activity, some of them showing concentration dependence. The majority of the samples were also able to inhibit lipid peroxidation. A strong correlation was observed between the results of DPPH and ILP assays and the flavonoids content of the samples. In the DNA protection assay, all the extracts were able to preserve DNA integrity. The antimycotic activity was performed against twelve fungi belonging to Basidiomycota and Ascomycota Divisions. *L. stoechas* subsp. *luisieri* exhibited the broadest activity spectra. *L. pedunculata* extracts were active against five fungi. *Cryptococcus neoformans* was the most sensitive, being inhibited by all the extracts. Our results led to the conclusion that *L. stoechas* subsp. *luisieri* and *L. pedunculata* can be useful as new sources of natural antioxidants and antimycotic agents, providing a possible valorization of the existing biodiversity and resources of Portuguese flora.

## 1. Introduction

Southwestern Iberia includes a peculiar and unique ecosystem, the* montado*, which combines agrosilvopastoral system with crop cultivation, maintaining high levels of biodiversity. It is protected under the Pan-European-Natura 2000 network [1, 2]. Many native species are found at the* montado*, including some endemics. All of them are adapted to very difficult edaphoclimatic conditions thus developing several functional and chemical features that allow their survival. Some of these plants have been used in traditional medicine and might provide bioactive natural compounds or extracts that may enable different approaches in the pharmaceutical area. This is the case of* Lavandula* genus (Lamiaceae) that comprises 39 species, some of them being used for centuries either dried or as essential oil for a variety of therapeutic, cosmetic, fragrance and condiment purposes. In addition to the essential oils with great economic value, several* Lavandula* species are also distinguished for their antioxidant potential and biological activities against the growth of a wide range of microorganisms [3–8].

The existence of eight species and subspecies of this genus is referenced in the Iberian Peninsula [9]. Some of them are found in the* montado *area or nearby, including* Lavandula stoechas *subsp.* luisieri *(Rozeira) Rozeira (Syn:* L. luisieri*;* L. stoechas *var.* luisieri*) and* Lavandula pedunculata *(Mill.) Cav. (Syn:* L. pedunculata *subsp.* sampaiana*).

*L. stoechas* subsp.* luisieri* is considered endemic in Iberia [9]. It is a perennial shrub that grows up to 20–60 cm, characterized by the opposite, narrow oblong-lanceolate gray leaves of different sizes and small lilac flowers arranged in dense short-stalked spikes (0–30 mm), all of them densely covered by an indumentum that includes several types of secretory hairs [10, 11].* L. pedunculata* is more commonly found throughout the Iberic Peninsula and can reach up to 70 cm tall. Its long-stalked spikes, full of lilac flowers, can reach 24 cm and are covered by different kinds of glandular trichomes [10, 11].

In order to make a step change on the conservation and valorization of the Portuguese* montado* flora, our goal is to study its potential through the botanical, phytochemical, and biological characterization of some selected plants. In previous work, we evaluated several* montado* plant species for their antimicrobial activity [6, 12]. In those studies, a total of forty extracts with different polarities and chemical compositions, were assessed against a panel of sensitive and resistant Gram positive and Gram negative bacteria, as well as* Mycobacterium smegmatis* and* Candida albicans*. Very interesting results were obtained with the most polar extracts of* P. broteroi*,* E. lusitanica*, and* Q. faginea*, when tested against sensitive and resistant* Staphylococcus aureus* strains [6]. In the present work, the essential oils and ten extracts of* Lavandula stoechas* subsp.* luisieri* and* Lavandula pedunculata*, were evaluated and compared for their* in vitro* antioxidant and antimycotic activities. The evaluation of the antioxidant activity was performed using chemical and biochemical experiments, namely, DPPH radical scavenging, inhibition of lipid peroxidation, and DNA protection assays. The antimycotic activity was performed by the broth microdilution method against twelve fungi species, chosen primarily on the basis of their importance as opportunistic pathogens of humans. Furthermore, the total phenolics and flavonoids content of essential oils and extracts were also determined in order to try to establish a correlation between the chemical composition and the biological activities.

## 2. Materials and Methods

### 2.1. Plant Material and Preparation of Samples

Aerial parts of* L. stoechas* subsp.* luisieri* and* L. pedunculata* were collected in 2010 during the flowering phase, from two study locations, at Southwestern and Center regions of Portugal, respectively. Plants were identified at the Lisbon Botanical Garden Herbarium (LISU) and Lisbon Agronomic Institute Herbarium (LISI), where vouchers were deposited. The plant material was dried in the shadow at room temperature and powdered before use. The crude extracts were prepared as previously reported [6]. Briefly, 100 g of powdered plant material were sequentially extracted with 300 mL of* n*-hexane (*n*-Hex), dichloromethane (CH_2_Cl_2_), ethyl acetate (EtOAc), methanol (MeOH) and water (H_2_O), during 24 h, at room temperature with occasional shaking. After filtration, the extracts were concentrated under reduced pressure at 40–45°C, and stored at −20°C until further use. Essential oils of the powdered air-dried aerial parts (150 g of each species) were obtained by hydrodistillation during 3 h, using a Clevenger apparatus, according to European Pharmacopoeia (Council of Europe, 2007). The essential oils were stored at −20°C until further use.

### 2.2. Phytochemical Analysis

#### 2.2.1. Evaluation of Total Phenolics Content

The concentrations of phenolic compounds were assessed using the Folin-Ciocalteu reagent according to the method described by Singleton and Rossi [13], with minor modifications. 100 *μ*L of extracts (2 mg/mL) were added to 0.2 mL of Folin-Ciocalteu reagent. After 3 min, 1 mL of Na_2_CO_3_ (15%) and 2 mL of water were added, and the mixture was kept in the dark at room temperature for 2 h. The absorbance was measured at 765 nm (Shimadzu UV-160 spectrophotometer). A calibration curve of gallic acid was prepared and the results were expressed as gallic acid equivalents (mg GAE/g extract) based on five points regression curve, 0.00–0.20 mg/mL,* R* ^2^ = 0.9983. Results are expressed as the mean ± SD from experiments performed in triplicate.

#### 2.2.2. Evaluation of Total Flavonoids Content

The flavonoids quantification was performed according to the technique presented by Zhishen et al. [14]. Briefly, 500 *μ*L of extracts (2 mg/mL), 2 mL of water and 150 *μ*L de NaNO_2_ 5% were mixed. After 5 min incubation at room temperature, in the dark, each sample was stirred with 150 *μ*L of AlCl_3_ 10%, 1 mL of NaOH 1 M e 2 mL of water. After 10 min incubation in the dark at room temperature the absorbance was measured at 510 nm (Shimadzu UV-160 spectrophotometer). Rutin was used as the standard for a calibration curve. The total flavonoids content was expressed in rutin equivalents (mg RE/g extract) based on four points regression curve, 0.005–0.080 mg/mL,* R* ^2^ = 0.9923. Results are expressed as the mean ± SD from experiments performed in triplicate.

### 2.3. Chemical and Biochemical Assays to Evaluate Antioxidant Potential

#### 2.3.1. DPPH Radical-Scavenging Activity

The assay was performed according to the method described by Sarikurkcu et al. [15]. A methanol solution (1.5 mL) of each sample at three different concentrations (50, 200, and 400 *μ*g/mL) was added to 9 mL of DPPH (in methanol, 60 mM). After vigorous shaking, the mixtures were incubated for 30 min in the dark. The absorbance was measured at 517 nm. For each dilution of the extracts, the DPPH scavenging activity (*I*, %) was determined according to the following expression:* I*(%) = 100 × [(*A*_blank_ − *A*_sample_)]/*A*_blank_, where *A*_blank_ is the absorbance of the DPPH (control reaction, containing all reagents except the test compound) and *A*_sample_ is the absorbance of the tested samples. BHT and ascorbic acid were used as positive controls. Results are expressed as the mean ± SD from experiments performed in triplicate.

#### 2.3.2. Inhibition of Lipid Peroxidation

This assay was performed using the method described by Liégeois et al. [16]. 30 *μ*L of linoleic acid dispersion (16 mM in borate buffer, pH 9), was added to 2.8 mL of phosphate buffer (0.05 M, pH 7.4). The oxidation was initiated with the addition of 150 *μ*L of AAPH (2,2′-Azobis(2-amidinopropane) dihydrochloride; 40 mM) solution and carried out with 20 *μ*L of sample (0.2 mg/mL). After incubation for 20 minutes in the dark at room temperature, the absorbance at 234 nm was measured. BHT was used as positive control (0.2 mg/mL). The percentage of lipid peroxidation inhibition (*I*, %) was calculated according to the following expression:* I*(%) = 100 × [(*A*_blank_ − *A*_sample_)]/*A*_blank_, where *A*_blank_ is the AAPH absorbance and *A*_sample_ is the absorbance of the tested samples. Results are expressed as the mean ± SD from experiments performed in triplicate.

#### 2.3.3. DNA Protection Assay

Antioxidant potential of plant extracts was evaluated as described by Inayatullah et al. [17]. Total DNA from* Escherichia coli* ATCC 25922 was isolated with the JetFlex Genomic DNA Purification Kit (Genomed) according to manufacturer's protocol. 5 *μ*L of DNA dissolving in buffer TE (10 mM Tris, 1 mM EDTA) was mixed with three different concentrations of extracts (10, 100 and 1000 ppm). Fenton reaction was induced by addition of H_2_O_2_ (45 *μ*L, 30%) and FeSO_4_ (3 *μ*L, 2 mM). Four controls (untreated DNA, DNA treated with FeSO_4_, DNA treated with H_2_O_2_, DNA treated with both FeSO_4_ and H_2_O_2_) were run simultaneously. Samples were incubated at 37°C in the dark for one hour. The reaction was stopped by addition of 2 *μ*L of 6x bromophenol blue. The samples were electrophoresed on a 1% agarose gel at 100 V and then were stained with the ethidium bromide solution and finally visualized in a gel documentation system (UVtec). Untreated DNA was used as positive control and revealed a band corresponding to the intact supercoiled DNA.

### 2.4. Evaluation of Antifungal Activity

#### 2.4.1. Microorganisms

*Candida* species (*C. albicans* (ATCC 90028),* C. dubliniensis* (FFUL 21),* C. glabrata* (FFUL 12B),* C. tropicalis* (ATCC 750),* C. guilliermondii* (FFUL 1403),* C. kruzei* (ATCC 6258) and* C. parapsilopsis* (ATCC 90018)),* Rhodotorula rubra* (FFUL190),* Trichosporon cutaneum* (FFUL 18H),* Saccharomyces cerevisiae* (FFUL 1997),* Cryptococcus neoformans* (FFUL 948), and* Aspergillus niger* (ATCC 16404).

#### 2.4.2. MIC Determination

The antifungal* in vitro* evaluation of microorganisms susceptibility was performed by the broth microdilution standardized method described by CLSI [18]. Briefly, a stock solution of the extracts in dimethyl sulfoxide was diluted with culture medium to a final concentration of 1 mg/mL. 98-wells microplates were filled with 100 *μ*L of RPMI medium. 100 *μ*L of the extract solutions were placed in the first well and serial two fold dilutions was performed. 10 *μ*L of a suspension of microorganisms (1.0 × 10^3^–1.5 × 10^3^ cells/mL) were added to each well and the plates were incubated for 24 h at 30°C. Ketoconazole and amphotericin B were used as positive controls. The minimal inhibitory concentration (MIC) was defined as the lowest concentration where no visible growth was observed.

### 2.5. Statistical Analysis

The data are expressed as means ± standard deviation (SD) and individual experiments were performed in triplicate. The statistical analysis was kindly performed by Professor Humberto Ferreira (DQFT, Faculty of Pharmacy, University of Lisbon). The analyses were done using SPSS version 21.0 software. One Way Analysis of Variance (ANOVA) coupled with the Tukey's post-hoc tests were used to compare the data and to identify means with significant differences. When non statistical differences were detected the *P* value was presented. Two-sided *P* values of *P* < 0.001 and *P* < 0.05 were considered significant.

## 3. Results and Discussion

### 3.1. Total Phenolics and Flavonoids Content

**Figure 1:**
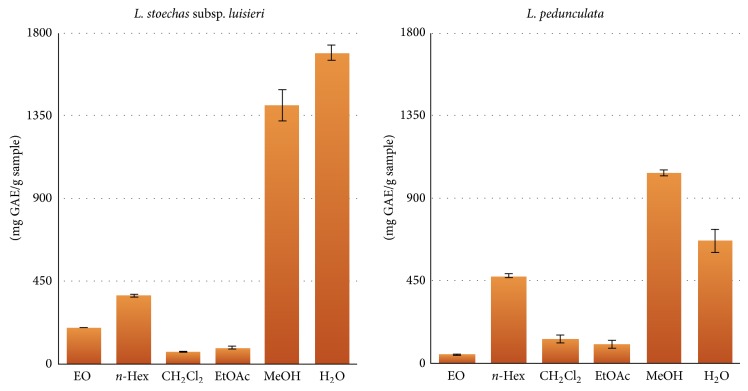
Total phenolics content (mg GAE/g sample) for* L. stoechas *subsp.* luisieri *and* L. pedunculata* essential oils (EO), and extracts [*n*-hexane (*n*-Hex), dichloromethane (CH_2_Cl_2_), ethyl acetate (EtOAc), methanol (MeOH) and water (H_2_O)].* L. stoechas *subsp.* luisieri* pairs of extracts CH_2_Cl_2_/EtOAc (*P* = 0.959) and EO/EtOAc (*P* = 0.058) displayed no statistically significant differences. Same statistical results were observed for EO/CH_2_Cl_2_/EtOAc (*P* = 0.055) from* L. pedunculata* extracts. For the remaining extracts *P* < 0.001.

**Figure 2:**
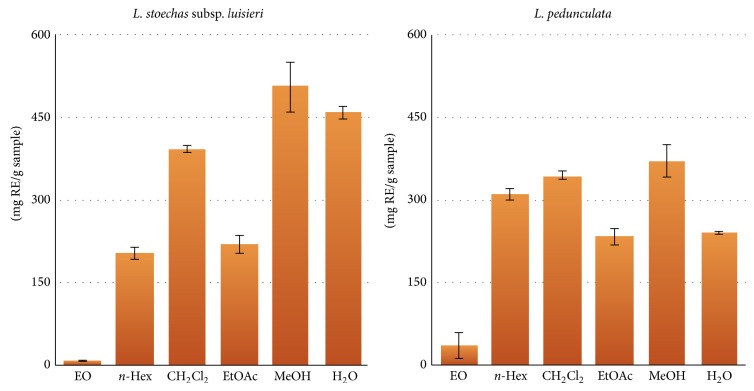
Total flavonoids content (mg RE/g sample) for* L. stoechas *subsp.* luisieri *and* L. pedunculata* essential oils (EO), and extracts [*n*-hexane (*n*-Hex), dichloromethane (CH_2_Cl_2_), ethyl acetate (EtOAc), methanol (MeOH) and water (H_2_O)]. For* L. stoechas *subsp.* luisieri*, no statistically significant differences were observed between the pairs* n*-Hex/EtOAc (*P* = 0.915) and MeOH/H_2_O (*P* = 0.135). Same statistical results were observed for EtOAc/H_2_O (*P* = 0.99),* n*-Hex/CH_2_Cl_2_ (*P* = 0.249) and CH_2_Cl_2_/MeOH (*P* = 0.459) from* L. pedunculata* extracts. For the remaining extracts *P* < 0.001.

Phenolic compounds and flavonoids are important secondary metabolites that are synthesized by plants during development. They possess an array of health-promoting benefits and have engaged a great deal of scientific interest due to their health promoting effects as antioxidants [19]. Phenolic compounds are ubiquitous in plants and they can act as chelators and free radical scavengers especially over hydroxyl, peroxyl, superanions, and peroxynitriles. Flavonoids are highly effective scavengers of most oxidizing molecules and of several free radicals related to various pathologies. They are also able to chelate metals and activate some antioxidant enzymes [20]. Therefore, it is important to correlate the total phenolics and flavonoids content with the antioxidant potential of each plant extract. The total phenolics content expressed as mg GAE/g extract and the total flavonoids content expressed as mg RE/g extract are shown in Figures 1 and 2, respectively.

As can be observed, for both* Lavandula* species, the concentration of total phenolics has a strong dependence on the solvent used on extraction. As expected, the amount of total phenolics was higher in polar fractions, namely in methanol and water extracts (Figure 1).* L. stoechas *subsp.* luisieri* extracts displayed a total phenolics content ranging from 67.4 ± 0.7 (CH_2_Cl_2_ extract) to 1689.3 ± 41.9 mg GAE/g (H_2_O extract). Comparatively,* L. pedunculata* extracts had lower content of total phenolics, which ranged from 50.0 ± 0.2 (essential oil) to 1040.3 ± 17.8 GAE mg/g (MeOH extract).

Concerning the total flavonoids content, it can be observed in Figure 2 that, excepting the essential oil, their distribution is more homogeneous among all the extracts of both species, probably due to the polarity of these compounds family. Comparing both plant extracts,* L. stoechas* subsp.* luisieri* had the highest content of flavonoids in the MeOH (506.2 ± 45.7 mg rutin/g) and H2O (458.6 ± 11.8 mg rutin/g) extracts, followed by the CH_2_Cl_2_ extract (392.3 ± 7.8 mg rutin/g). Regarding* L. pedunculata*,* n*-Hex, CH_2_Cl_2_ and MeOH (310.7 ± 10.6, 344.7 ± 8.8 and 371.9 ± 29.8 mg rutin/g, resp.) were the extracts that displayed high levels of flavonoids.

### 3.2. Antioxidant Activity

Oxidants or reactive oxygen species (ROS) includes radicals and chemical species that are produced by living organisms during normal oxidative processes (e.g., production of energy, metabolic regulation and signal transduction) or as an answer to stress situations like temperature or light radiation. If there is an imbalance between ROS and antioxidant defense molecules, several diseases might succeed including atherosclerosis, carcinogenesis, neurodegenerative and chronic inflammatory pathologies [21]. Antioxidants are molecules that play an important role on scavenging the ROS species, preventing the initiation chain, acting on the catalytic systems that neutralized ROS (enzymes) or inactivating metal ions [22]. Based on ROS inactivation mechanism, most of the* in vitro* antioxidant assays have been divided in two major groups: (1) hydrogen atoms transfer (HAT) and single electron transfer (ET) reactions based assays. In this work and according to these data, two methods for evaluating the antioxidant activity of* Lavandula* species were selected: the DPPH assay that is included in the ET techniques and the inhibition of lipid peroxidation assay, a HAT method [22]. According to Ou et al. [23], the HAT plays a dominant role in biological redox mechanisms, competing* in vivo *with the ET reactions.

**Figure 3:**
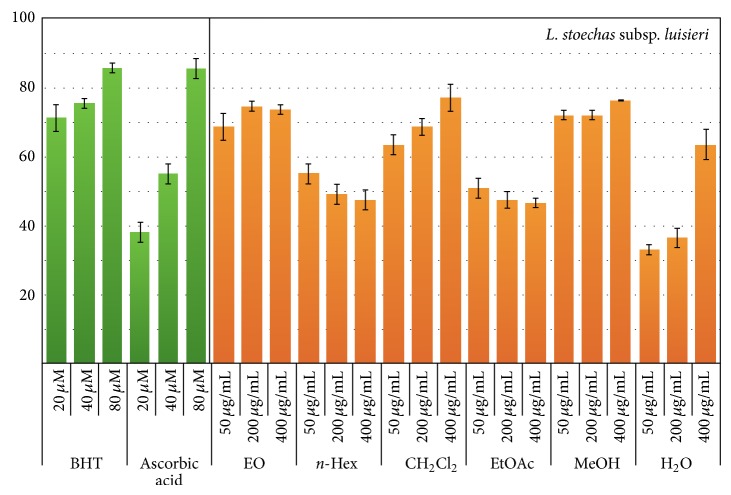
DPPH radical scavenging activity (%) of* L. stoechas *subsp.* luisieri *essential oil (EO; *P* = 0.121) and extracts [*n*-hexane (*n*-Hex; *P* = 0.018), dichloromethane (CH_2_Cl_2_; *P* < 0.001), ethyl acetate (EtOAc; *P* = 0.296), methanol (MeOH; *P* = 0.336), and water (H_2_O; *P* < 0.001)].

**Figure 4:**
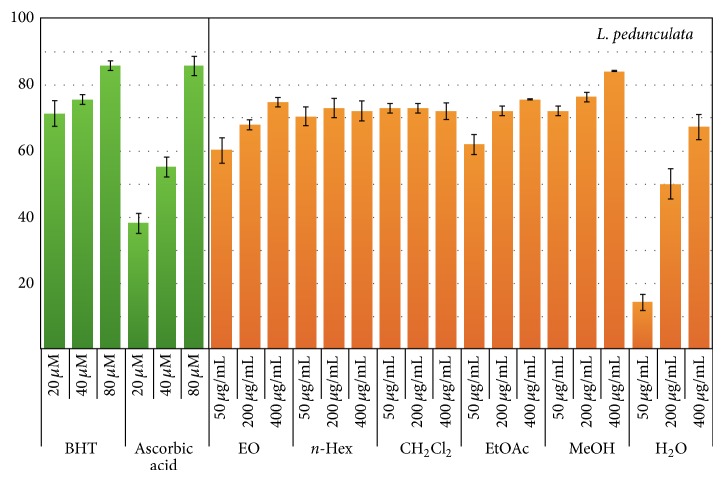
DPPH radical scavenging activity (%) of* L. pedunculata* essential oil (EO; *P* < 0.001) and extracts [*n*-hexane (*n*-Hex; *P* = 0.317), dichloromethane (CH_2_Cl_2_; *P* = 0.630), ethyl acetate (EtOAc; *P* < 0.001), methanol (MeOH; *P* < 0.003), and water (H_2_O; *P* < 0.001)].

The DPPH radical-scavenging activity of the ten extracts and essential oils of* L. stoechas *subsp.* luisieri *and* L. pedunculata* were determined and the results are shown in Figures 3 and 4. BHT and ascorbic acid were used as positive controls (20, 40 and 80 *μ*M). Three concentrations were assayed for each extract (50, 200 and 400 *μ*g/mL). All samples showed radical scavenging activity. Some of them also showed scavenging activity concentration dependence (EO, CH_2_Cl_2_, MeOH, and H_2_O extracts from* L. stoechas *subsp.* luisieri*, and EO, EtOAc, MeOH, and H_2_O extracts from* L. pedunculata*). When tested at 50 *μ*g/mL, the EO (68.7 ± 2.9%), CH_2_Cl_2_ (63.6 ± 1.5%), and MeOH (72.0 ± 4.4%) extracts from* L. stoechas *subsp.* luisieri*, presented higher DPPH radical scavenging activity, comparable to those of ascorbic acid (55.1 ± 2.9% at 40 *μ*g/mL) and BHT (75.4 ± 1.5% at 40 *μ*g/mL). Similarly, when tested at 50 *μ*g/mL, all extracts from* L. pedunculata* (with the exception of H_2_O extract) showed DPPH radical scavenging activity values between 60.2 ± 1.5% and 72.3 ± 1.5%. Curiously, the H_2_O extracts from both* Lavandula* species only exhibited significant DPPH radical scavenging activity values when tested at 400 *μ*g/mL (68.9 ± 2.9%).

The results of DPPH scavenging assay revealed, for almost all the data, a very good correlation with the flavonoids content, displaying the* L. pedunculata* extracts the highest correlation. Nevertheless, it must be noticed that other compounds like terpenes, alkenes, carbonyl compounds (constituents for instance of the essential oil), lignans, and anthocyanines, among other compounds, may be related to the scavenging activity and their presence was not evaluated in this work.

**Figure 5:**
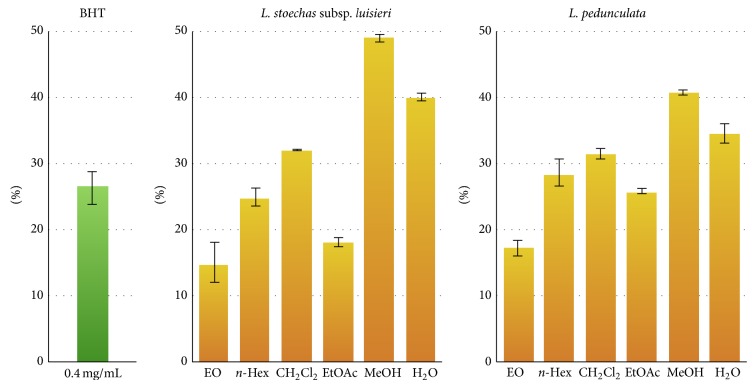
Inhibitory effects (%) of* L. stoechas *subsp.* luisieri *and* L. pedunculata* essential oils and extracts on lipid peroxidation. For* L. stoechas *subsp.* luisieri* no statistically significant differences were observed between EO/EtOAc (*P* = 0.154) extracts. Same statistical results were observed for* n*-Hex/EtOAc (*P* = 0.150),* n*-Hex/CH_2_Cl_2_ (*P* = 0.119) and CH_2_Cl_2_/H_2_O (*P* = 0.080). For the remaining extracts *P* < 0.001.

The antioxidant activity of the extracts and essential oils of* L. stoechas *subsp.* luisieri *and* L. pedunculata* was also evaluated measuring the inhibition of oxidation of the exogenous linoleic acid by a thermal free radical producer (AAPH). All samples were tested at 0.2 mg/mL. BHT was used as positive control (0.2 mg/mL). From Figure 5, it was evident that, excepting the* L. stoechas *subsp.* luisieri *and* L. pedunculata* essential oils (14.8 ± 3.1% and 17.2 ± 1.3%, resp.) and the EtOAc extract from* L. stoechas *subsp.* luisieri *(18.1 ± 0.8%), all the tested extracts displayed similar or higher lipid peroxidation inhibition activity than BHT (22.6 ± 2.5%). The MeOH and H_2_O extracts of both species exhibited the highest % inhibition capacity with values that range between 34.6 ± 1.5% and 49.1 ± 0.7%. It is interesting to note that the highest lipid peroxidation inhibition activity observed in the most polar extracts were correlated with those that displayed higher phenolics and flavonoids content (Figure 2).

**Figure 6:**
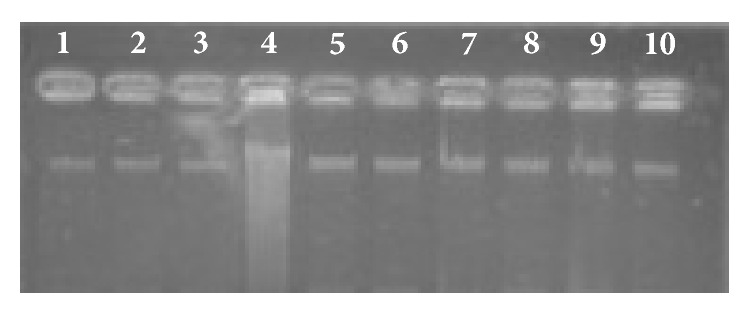
Protection induced by* L. pedunculata* and* L. stoechas *subsp.* luisieri *methanol extracts on DNA nicking caused by hydroxyl radicals. Lanes 1–4 controls (1: DNA; 2: DNA + FeSO_4_; 3: DNA + H_2_O_2_; 4: DNA + FeSO_4_ + H_2_O_2_); lanes 5–7:* L. pedunculata* (5: DNA + FeSO_4_ + H_2_O_2_ + MeOH extract 10 ppm; 6: DNA + FeSO_4_ + H_2_O_2_ + MeOH extract 100 ppm; 7: DNA + FeSO_4_ + H_2_O_2_ + MeOH extract 1000 ppm), lanes 8–10:* L. stoechas *subsp.* luisieri *(8: DNA + FeSO_4_ + H_2_O_2_ + MeOH extract 10 ppm; 9: DNA + FeSO_4_ + H_2_O_2_ + MeOH extract 100 ppm; 10: DNA + FeSO_4_ + H_2_O_2_ + MeOH extract 1000 ppm).

The antioxidant potential of essential oils and extracts was further investigated by the protection of DNA against damage induced by Fenton reaction (Figure 6). This assay aims to evaluate the ability of the extracts to prevent the DNA degradation induced by the hydroxyl radicals formed after the addition of FeSO_4_ and H_2_O_2_. Hydroxyl radicals from the Fenton reagent are able to react with the nucleotides in DNA and cause strand breakage, which can have an important role in many biological processes including aging, and carcinogenesis [24]. Although all the ten extracts and the essential oils were assayed at three different concentrations (10, 100 and 1000 ppm), the results obtained were rather similar to those displayed in Figure 6, which exhibit the effects on DNA protection obtained with the MeOH extracts of* L. stoechas *subsp.* luisieri *(lanes 5–7) and* L. pedunculata *(lanes 8–10). As can be observed, the supercoiled DNA appears as a predominant and well defined band (lane 1) and no alteration were induced by the addition of FeSO_4_ (lane 2) or H_2_O_2_ (lane 3). When both reagents are present along with the DNA, a random cleavage is induced by the hydroxyl radicals being that phenomenon evident by the smear observed in lane 4. No apparent DNA damage was observed in any of the tested samples (lanes 5 to 10). In conclusion, there is evidence that all samples exhibited significant DNA protection activity and were able to preserve DNA integrity.

It is noteworthy that* L. angustifolia* is the only* Lavandula *specie included in pharmaceutical formulations [25]. The antioxidant studies on the essential oils and extracts of this specie have shown discordant results. Some authors reported no activity [26, 27] while others stated the existence of antioxidant activity [28]. However, all of them sustained a modest role as antioxidants, less potent than other members of the Lamiaceae family. The inconsistencies between those observations and the results presented in this work might be related to differences observed on the chemical composition of* Lavandula* species chemotypes. Several authors have already pointed out the fact that differences in the yield and in the chemical composition of the essential oils and extracts of aromatic species are common. These differences should be probably due to several conditions, particularly the genotype, the environment, the geographic origin, the period of harvest, the conditions of drying and the method of extraction including the scientific equipment used [5, 29–31].

### 3.3. Antimycotic Activity

To perform the antimycotic assays twelve species were selected. Fungi used in this study were chosen primarily on the basis of their importance as opportunistic pathogens of humans.* Candida *spp. and* Cryptococcus *spp. are the major responsible for the nosocomial fungal infections according to National Nosocomial Infection Surveillance [32].* Aspergillus niger* is a cosmopolitan fungus responsible for the production of mycotoxins that are hepatogenic and nephrogenic [33].* Rhodotorula* and* Trichosporon *spp. are emerging opportunistic pathogens, particularly in haematological, immunosuppressed and/or cancer patients [34, 35].* Saccharomyces cerevisiae* is the most well characterized eukaryote and is unique in its broad applications [36].

The results are summarized in Table 1. It should be pointed out that a quite narrow endpoint criterion for MIC was considered and only crude extracts with MIC values lower than 100 *μ*g/mL were considered to be active [37, 38]. The MIC endpoint criterion for* in vitro* testing of plant extracts is not consensual and several authors assume a much higher cut-off value [32, 39, 40]. As displayed in Table 1, all the extracts were active on one (*L*.* pedunculata* MeOH and EtOAc extracts) or more fungi species.* L. stoechas* subsp.* luisieri *exhibited the broadest activity spectra presenting MIC values ranging from 7.5 to 62.5 *μ*g/mL against seven out of twelve fungi species (*A. niger*,* C. albicans*,* C. guilliermondii*,* S. cerevisiae*,* C. neoformans*,* R. rubra*, and* T. cutaneum*). All the* L. stoechas* subsp.* luisieri *extracts were active on* C. neoformans* and* S. cerevisiae*, presenting MIC values between 15.5 and 31. It is noticeable the activity of essential oil and CH_2_Cl_2_ extract of* L. stoechas *subsp.* luisieri*, which inhibited the growth of four fungi including* C. albicans*, one of the most common human pathogens.

*L. pedunculata* extracts were active on five species, namely* C. guilliermondii*,* C. neoformans*,* R. rubra*,* S. cerevisiae*, and* T. cutaneum* (MIC 15.5–62.5 *μ*g/mL). The most active against these fungi was the* n*-Hex extract (MIC 31–62 *μ*g/mL).* C. neoformans* was the most sensitive, being inhibited by all the extracts. None of the tested extracts were able to inhibit the growth of* C. dubliniensis*,* C. glabrata*,* C. krusei*,* C. parapsilosis* and* C. tropicalis*.

In general, it can be pointed out that the nonpolar extracts (essential oils,* n*-Hex, CH_2_Cl_2_) were more active than the polar ones (AcOEt, MeOH and H_2_O). From our results it can be observed that fungi belonging to Basidiomycota Division are much more sensitive to the tested extracts than the species included in the Ascomycota Division, especially against* C. neoformans*. Cryptococcosis is until now a worldwide mycose that has been characterized by the development of resistance among many strains after antifungal therapies. Therefore, alternatives should be considered. This different activity observed against the species tested could be explained because these species are phylogenetically different, and the difference of fungi nature, the variation of metabolism trend, the wall organization, and the dimorphism are other factors that can affect the resistance of some species against antifungal agents comparatively to others.

Zuzarte et al. [41, 42] reported the antifungal activity of the essential oils of both* Lavandula *species presented in this work, but not from our places of collection. Nevertheless, the results are not superimposed and this is related with the different composition of the essential oils. Often the species show different qualitative and quantitative composition, being the relative amounts of their main constituents altered, and so, changing their physical and biological characteristics and properties, which correspond to different chemotypes [43]. Feijão et al. [44, 45] identified the essential oils components of the same species and populations used in the present study, through GCMS technique.* L. pedunculata* chemotype had fenchone as major component (almost 80%) [44], whereas Zuzarte et al.'s [41] chemotype presented only at maximum 45% of this compound. Similar conclusions were observed on chemical composition of* L. stoechas *subsp.* luisieri* essential oil. The chemotype studied in this work displayed not only different percentages of some constituents (limonene, camphor, *α*-pinene, 1,8-cineole and fenchone) but also exhibited some components like pulegone or 5-methylene-2,3,4,4-tetramethyl cyclopentenone that were absent in the Zuzarte et al.'s essential oils [42, 45].

## 4. Conclusions

Although in the past decades the chemical and bioactivity of* Lavandula* essential oils have been the midpoint of several published studies, minor attention has been handed out to the extracts of these plants. As part of our ongoing research on the properties and possible applications of the Portuguese* montado* flora, we investigated the antioxidant and antimycotic activities of two* Lavandula* species, namely* L. stoechas *subsp.* luisieri* and* L. pedunculata*. The results demonstrated that both species displayed a high content of phenolic and flavonoid compounds. Particularly, flavonoids had a great contribution to the antioxidant activity, showing a high correlation with the results obtained in the DPPH scavenging and inhibition of lipid peroxidation assays. However, the existence of other compounds with antioxidant properties cannot be disregarded. From the best of our knowledge the DNA protection assay was never performed in these taxa, neither with the extracts nor the essentials oils. In this work, it was established that the samples exhibited a very important effect of protecting the* in vitro* DNA damage induced by hydroxyl radicals, therefore being able to preserve DNA activity. Concerning the antimycotic assays, promising results were also obtained with the essential oils and the nonpolar extracts of both plants, when tested against several selected species belonging to Ascomycota and Basidiomycota Divisions.

In conclusion,* L. stoechas *subsp.* luisieri* and* L. pedunculata* can be useful as a new potential source of natural antioxidants and antimycotic agents and can be directly applied on the composition of dermocosmetic formulations as antiaging products or, as raw material for the isolation of bioactive chemical compounds. Ultimately, this could contribute to the valorization of the existing biodiversity and resources of Portuguese native species of* Lavandula*, promoting its sustainable use and producing novel economic revenues.

## Figures and Tables

**Table 1:** Minimal inhibitory concentration (MIC) of *Lavandula stoechas *subsp. *luisieri* and *Lavandula pedunculata* essential oils and extracts against clinically important fungi species.

Fungi	MIC values (*µ*g/mL)													
	*L. stoechas *subsp. *luisieri* samples						*L. pedunculata* samples						Controls	
	EO	*n*-Hex	CH_2_Cl_2_	EtOAc	MeOH	H_2_O	EO	*n*-Hex	CH_2_Cl_2_	EtOAc	MeOH	H_2_O	Amp. B	Ket.
*Aspergillus niger *	**15.5**	**7.5**	**31**	>100	**62.5**	>100	>100	>100	>100	>100	>100	>100	0.47	0.064
*Candida albicans *	>100	>100	**62.5**	>100	>100	>100	>100	>100	>100	>100	>100	>100	0.125	2
*Candida guillermondii *	**62.5**	>100	>100	>100	>100	>100	**62.5**	**62.5**	>100	>100	>100	>100	0.064	14
*Cryptococcus neoformans *	**15.5**	**31**	**31**	**31**	**62.5**	**62.5**	**15.5**	**31**	**15.5**	**15.5**	**15.5**	**62.5**	0.125	6
*Rhodotorula rubra *	>100	>100	>100	**31**	>100	>100	**62.5**	**62.5**	**62.5**	>100	>100	>100	0.094	2
*Saccharomyces cerevisiae *	**31**	**62.5**	**62.5**	**62.5**	**31**	**62.5**	>100	**31**	**31**	>100	>100	**62.5**	0.016	0.5
*Trichosporon cutaneum *	**31**	>100	>100	>100	**62.5**	**62.5**	**62.5**	**31**	**31**	>100	>100	>100	0.25	0.2

All the tested extracts were considered inactive against *Candida dubliniensis*, *Candida glabrata*, *Candida kruzei*, *Candida parapsilosis*, and *Candida tropicalis* (MIC values >100 *µ*g/mL). Positive controls: Amp. B: amphotericin B; Ket: ketoconazole. EO: essential oil; *n*-Hex: *n*-hexane; CH_2_Cl_2_: dichloromethane; EtOAc: ethyl acetate; MeOH: methanol; H_2_O: water.
